# ID 50 - Eficácia e Segurança do Apremilaste em Monoterapia para Adultos com Psoríase em Placas Moderada a Grave: uma revisão sistemática e metanálise de ensaios clínicos randomizados

**DOI:** 10.5327/2237-9622.2025.v34s1.50

**Published:** 2025-11-25

**Authors:** Franciele Iachecen, Adalton Ribeiro, Iara Bernz

**Keywords:** psoríase em placas moderada a grave, apremilaste, metanálise

## Abstract

**Introdução:**

A psoríase é uma doença crônica de natureza inflamatória e imunomediada, que compromete a pele e as articulações, sendo a psoríase em placas o fenótipo clínico mais prevalente. Recentemente, o apremilaste, um inibidor oral da fosfodiesterase tipo 4, foi aprovado para o tratamento da psoríase em placas, destacando-se como uma nova alternativa terapêutica. Esta revisão sistemática teve como objetivo primordial avaliar a eficácia e a segurança do apremilaste como monoterapia para o tratamento da psoríase em placas de moderada a grave.

**Método:**

Foram incluídos nesta revisão ensaios clínicos randomizados que compararam o apremilaste, nas dosagens de 30 mg e 20 mg, ambas administradas duas vezes ao dia, com placebo, visando determinar a eficácia terapêutica do apremilaste em pacientes com psoríase em placas moderada a grave. A pesquisa bibliográfica foi conduzida nas bases de dados PubMed, Embase e CENTRAL. Os desfechos primários avaliados incluíram o PASI-75 e a resposta da Avaliação Global Estática do Médico (sPGA). Desfechos secundários avaliaram a eficácia do apremilaste em relação ao placebo e, adicionalmente, foram considerados os eventos adversos associados ao tratamento, avaliando-se sua frequência e gravidade em comparação ao placebo.

**Resultados:**

Foram considerados elegíveis para a análise nove ensaios clínicos randomizados, envolvendo um total de 3.257 participantes. O apremilaste, administrado nas dosagens de 30 mg e 20 mg, duas vezes ao dia, demonstrou uma eficácia significativamente superior ao placebo na obtenção do PASI-75 ao longo de 16 semanas de tratamento (RR = 4,23; IC 95%: 3,29 - 5,43 e RR = 3,20; IC 95%: 2,00 - 5,14, respectivamente), bem como na Avaliação Global Estática do Médico (sPGA) (RR = 3,90; IC 95%: 3,14 - 4,86 e RR = 2,21; IC 95%: 1,30 - 3,76, respectivamente).

**Conclusão:**

Os achados desta metaanálise indicam que a monoterapia com apremilaste constitui uma intervenção terapêutica eficaz para o manejo da psoríase em placas de moderada a grave, apresentando um perfil de segurança e tolerabilidade considerado aceitável.

